# Determination of drug-related problems according to PAIR criteria in dialysis patients: a cross-sectional study in tertiary care hospital

**DOI:** 10.1186/s40360-024-00754-6

**Published:** 2024-04-18

**Authors:** Aysel Pehlivanli, Sayeste Akkan Eren, Sule Sengul, Bilgen Basgut, Sehsuvar Erturk, A. Tanju Ozcelikay

**Affiliations:** 1 Faculty of Pharmacy, Department of Pharmacology, Baskent University, Ankara, Turkey; 2 Faculty of Pharmacy, Department of Clinical Pharmacy, Ankara University, Ankara, Turkey; 3 Department of Nephrology, Ankara University, School of Medicine, Ankara, Turkey; 4 Faculty of Pharmacy, Department of Pharmacology, Ankara University, Ankara, Turkey

**Keywords:** Chronic kidney disease, PAIR criteria, PCNE, Drug-related problem, Validation study

## Abstract

**Background:**

Dialysis patients are at high risk for drug-related problems (DRPs), which have significant consequences for their morbidity, mortality, and quality of life. Improved clinical outcomes can be achieved by preventing, identifying, and resolving these problems.

**Methods:**

This is a retrospective observational study. In this study, the PAIR instrument (Pharmacotherapy Assessment in Chronic Renal Disease) was validated for use in Turkish. Validation consisted of three stages: translation back-translation with expert panel evaluation, reliability analysis using the test-retest method, and conceptual validity with both Pharmaceutical Care Network Europe (PCNE) and PAIR used to determine DRPs prevalence.

**Results:**

In total, 104 patients (mean ± SD age, 54.1 ± 15.8 years; 53.8% male) were included in the study. An expert panel evaluated the items in the criterion based on their intelligibility, service of purpose, differentiation, and cultural suitability during the translation stage. Content validity index (CVI) score was found to be 0.95. The reliability analysis was performed by applying the test-retest method and calculating correlation coefficient on 30 randomly selected patients one month later. Correlation coefficient (p) was found to be 0.8. To evaluate conceptual validity, 104 patients’ pharmacotherapy plans were assessed using both the PAIR and PCNE criteria. The prevalence of DRPs according to PAIR criteria (100.0%) and PCNE (73.1%) were statistically significantly different (*p* < 0.001).

**Conclusions:**

As a result, PAIR criteria can identify clinically relevant DRPs in patients with CKD and is a new, validated tool to be used in Turkey, but may not be adequate for patients receiving dialysis. Therefore, it needs to be reviewed and updated for dialysis patients.

## Introduction

Chronic kidney disease (CKD) is estimated to be the fifth largest cause of life-year loss worldwide by 2040 [1, 2]. In 2021, the incidence of end-stage kidney disease (ESKD) requiring renal replacement therapy (RRT) in Turkey was calculated as 149.5 per million population (pmp). The annual incidence of ESKD requiring RRT is found to be 112.4 pmp for hemodialysis (HD), 15 pmp for peritoneal dialysis (PD), and 22.3 pmp for transplantation [3].

A major risk factor for CKD is diabetes mellitus (DM) and hypertension (HT), which account for two-thirds of all cases. Most of the patients in this population are elderly and suffer from multiple comorbidities, requiring multiple medications to be taken continuously [4–6]. According to previous studies, patients with CKD take an average of 8 medications (with polypharmacy prevalence increasing from 80 to 86% between stages 1 and 3 of CKD), while dialysis patients take 10 to 12 medications per day [7, 8]. A patient with advanced kidney disease may be more susceptible to drug-related problems (DRPs) associated with polypharmacy and changes in pharmacokinetics and pharmacodynamics [9].

At all stages of CKD, there is evidence that DRP is common in patients [10, 11]. Therefore, early identification of DRP in this population may contribute to improved survival, reduced disease progression, and reduced cardiovascular morbidity, thus contributing to cost reductions in health care [12, 13].

There are many tools used to detect DRPs. A significant part of these are implicit criteria based on the clinical knowledge of the practitioner [14]. DRPs can be detected by practitioners of all degrees quickly when explicit criteria are used [15, 16].

Pharmacotherapy Assessment in Chronic Renal Disease (PAIR) explicit criteria was developed in Canada to evaluate pharmacotherapy in patients with chronic kidney disease. The PAIR criteria assist in preventing, detecting, and managing DPR in individuals with CKD who are undergoing conservative treatment [17]. The DRP rate was found to be 21% and 80% in two studies using the PAIR criteria in CKD patients [17, 18].

### Aim

The primary aim of this study is to determine the validity and reliability results of the Turkish version of the PAIR criteria, which was developed to evaluate the pharmacotherapy plan of CKD patients. A second aim is to determine the prevalence and type of drug-related problems using the PAIR criterion in the treatment of these patients.

### Ethics approval

This study was performed in line with the principles of the Declaration of Helsinki. Approval was granted by the Ethics Committee of the University Ankara (Date 17.06.2021 /No İ6-409-21).

## Method

### Study design and setting

This retrospective study was conducted in the Nephrology Department of Ankara University School of Medicine, Ibn-i Sina Hospital. The hospital is a 1000-bed tertiary care hospital, and 34 are in the Nephrology service staffed by 10 nephrologists and 17 nurses. Also, there are outpatient hemodialysis and peritoneal dialysis units where approximately 150 patients are included.

### Inclusion criteria

- Male and female patients aged 18 years and older,
- Hemodialysis and peritoneal dialysis outpatients,
- Patients using at least 1 drug,
- With or without comorbidity, were included in the study.

### Exclusion criteria

- Patients with incomplete files or missing information were not included.

### Sample size and study population

The number of patients (n) to be included in the sample of the study was calculated as at least 91 patients when calculated with a 95% confidence interval, 5% margin of error, and 21% prevalence of dialysis [18] using the Raosoft® sample calculation program [19].

### Study process

The permission was obtained from Lyne Lalonde, the corresponding author of the article containing the PAIR criterion, provided that the original article is referenced.

### Translation stage

Translation of the scale into the Turkish language by 2 native Turkish speakers (1 pharmacologist and 1 clinical pharmacist), without making any changes on the scale by forward–backward translation procedure. In detail, the PAIR translation was performed independently by two professionals (1 pharmacologist (ATO) and 1 clinical pharmacist (AP)) fluent in the source language and culture, considering conceptual equivalence and avoiding literal translation. Following the synthesis, committee members assessed any linguistic, conceptual, and contextual discrepancies in a single version of the tool. This tool translated from the source language (British English) from a single version was used to assess semantic equivalence - the meaning of words or sentences in Turkish culture - and item equivalence from the original text. Also, the expert panel (1 nephrologist (SS) and 1 pharmacologist (BB)) was asked to evaluate the items in the criterion in terms of intelligibility, serving the purpose, distinguishing and cultural suitability by using the translation evaluation form, and expressing their opinions by evaluating the measurement level of each item by 1–4 points. In the evaluation to be made regarding the intelligibility of each question; 1 point is “not appropriate”, 2 points are “somewhat appropriate, the item needs to be adjusted”, 3 points are “quite appropriate but minor changes are necessary”, 4 points are “very appropriate” [20]. These people included in the expert panel are researchers who are interested in chronic kidney disease and follow-up patients (SS) and researchers who have previously conducted studies with a similar methodology (BB). The Content Validity Index (CVI) is calculated with the percentage of agreement between the views. As a result of the answers from the experts, each item getting 3 or 4 points above 80% is interpreted as a good CVI score [21]. To apply the first version of the tool, a group of Turkish professionals (10 patients who used drugs and were evaluated by the clinical pharmacist (AP)) had to conduct a pre-test. The tool’s adequacy, structure, and usefulness were to be verified. All operational difficulties were noted and discussed for each tool item with an expert panel. As a result of feedback from the expert panel (SS, BB), minor changes were made to the tool in version 2.

### Reliability analysis stage

The pharmacotherapy of 30 randomly selected patients will be re-evaluated by the clinical pharmacist 1 month later, the test-retest method will be applied, and the correlation coefficient (p) will be calculated. According to Landis & Kock’s criteria (1977), the closer to 1 the value, the greater the likelihood of agreement [22].

### Conceptual validity stage

Instrument validity refers to the ability to measure exactly what it was designed to measure. When an instrument’s conceptual coverage for a specific construct is subjectively judged, it is said to be conceptual [23]. The pharmacotherapy plan of all patients is evaluated without using the PAIR criteria. Additionally, unlike other studies, DRPs were evaluated using the PCNE V9.1, and whether there is a statistically significant difference between the number of drug-related problems detected is analyzed using a t-test.

### Data collection and statistical analysis

The data were collected from the patient’s medical records. Quantitative data were expressed as mean, standard deviation, median, maximum, and lowest values, percentages, and qualitative data were expressed as numbers and percentages in the statistical analysis to be used in the study. The normality of the data was determined by using Shapiro Wilk test. Between-group differences were analyzed using the Chi-square test with Fisher’s exact adjustment where appropriate for categorical variables and the t-test for continuous variables. Statistical significance was expressed as p < 0.05. IBM SPSS v23.0 software was used to evaluate the data.

## Results

In total, 104 patients (mean ± SD age, 54.1 ± 15.8 years; 53.8% male) were included in the study. The percentages of patients based on HD and PD were 53.8% (n = 56), and 46.2% (n = 48), respectively. The most common comorbidities of the patients were hypertension (n = 66, 63.5%), diabetes mellitus (n = 24, 23.1%), and cardiovascular diseases (15.4%). Most patients (n = 39, 37.5%) had only 1 comorbid disease. Most of the patients (n = 69, 66.3%) were using between 6–10 medications (Table 1).

**Table 1:** Demographic and clinical characteristics of the patients (N = 104)

Characteristics	N (%)
**Gender**	
Female	48 (46.2)
**Age**	
< 65	69 (66.3)
≥ 65	35 (33.7)
**Dialysis type**	
HD	56 (53.8)
PD	48 (46.2)
**ESKD etiology**	
Hypertensive nephropathy	21 (20.2)
Diabetic nephropathy	19 (18.3)
Polycystic kidney diseases	3 (2.9)
Glomerulonephritis	2 (1.9)
Obstructive nephropathy	2 (1.9)
Amyloidosis	1 (0.9)
Miscellaneous	24 (23.1)
Unknown etiology	32 (30.8)
Comorbidities	
Hypertension	66 (63.5)
Diabetes	24 (23.1)
Cardiovascular disease	16 (15.4)
Dyslipidemia	11 (10.6)
**Number of comorbidities**	
0	23 (22.1)
1	39 (37.5)
2	19 (18.3)
3 or more	23 (22.1)
**Number of drugs**	
1–5	22 (21.2)
6–10	69 (66.3)
11–15	13 (12.5)
**Laboratory findings, mean ± SD**	
Glucose (mg/dL)	127.5 ± 110.9
Bicarbonate (mmol/L)	21.7 ± 4.1
Hb (g/dL)	11.2 ± 1.7
HbA1c (%)	6.7 ± 1.9
Ferritin (ng/mL)	580.3 ± 725.0
Transferrin (mg/dL)	30.5 ± 15.6
Potassium (mmol/L)	4.2 ± 0.7
Phosphorus (mg/dL)	5.0 ± 1.4
Calcium (mg/dL)	8.8 ± 0.7
Vitamin D (nmol/L)	11.5 ± 7.0
Parathormone (pg/mL)	481.7 ± 469.9
LDL-cholesterol (mg/dL)	92.6 ± 37.2

*ESKD* End Stage Kidney Disease, *Hb* Hemoglobin, *LDL* Low-density lipoprotein, *SD* Standard deviation

The most prescribed drugs were calcium carbonate/acetate (64/791, 8.1%), sodium bicarbonate (62/791, 7.8%), darbepoetin (49/791, 6.2%), proton pump inhibitors (38/791, 4.8%), acetylsalicylic acid (37/791, 4.7%), lercanidipine (35/791, 4.4%), and cholecalciferol or vitamin D (35/791, 4.4%).

### Prevalence of DRPs

In this study, 495 DRPs were found, with an average of 4.8 ± 1.2 DRP per patient according to PAIR criteria. All patients had at least 1 DRP. The most prevalent categories of DRPs were “clinically significant DRPs not requiring a pharmaceutical intervention for patient follow-up” (95.8%), “interaction and drug taken inadequately” (3.0%), “inadequate use (inappropriate dosage or contraindicated agent)” (0.8%) (Table 2).

**Table 2:** Prevalence of DRP according to PAIR criteria for 104 CKD patients (N = 495)

Drug-related problem	N (%)
Inadequate use (inappropriate dosage or contraindicated agent)	4 (0.8)
The patient is receiving a medication that is not indicated, a non-steroidal anti-inflammatory	2 (0.4)
The patient is receiving too high a dose of pregabalin	1 (0.2)
The patient is receiving too high a dose of fenofibrate nanocrystals	1 (0.2)
Interaction and drug taken inadequately	15 (3.0)
The patient is experiencing a drug interaction between calcium and iron P.O. taken concomitantly	8 (1.6)
The patient is experiencing a drug interaction between his phosphate binder (calcium carbonate, sevelamer or lanthanum and levothyroxine)	7 (1.4)
Problems related to an over-the-counter medication or a natural health product	2 (0.4)
The patient is taking a purgative not indicated for kidney.	2 (0.4)
Clinically significant DRPs not requiring a pharmaceutical intervention for patient follow-up in a multidisciplinary predialysis clinic	474 (95.8)
The patient needs a drug treatment, bicarbonate of soda, because he has metabolic acidosis (HCO3 < 20 mmol/L) and does not present any contra-indications to bicarbonates	22 (4.4)
The patient needs a drug treatment, a hematopoietic agent, because his hemoglobin < 100 g/L and all other causes of anemia have been eliminated, but he is not receiving it	26 (5.3)
The patient needs a drug treatment, a statin, to treat his dyslipidemia (LDL > 2.0 mmol/L) and for appropriate cardiovascular prevention, but he is not receiving it	73 (14.7)
A patient with non-diabetic neuropathy whose urinary albumin/urinary creatinine ratio is > 200 mg/g, needs drug treatment (angiotensin-converting enzyme inhibitor or angiotensin receptor blocker) to slow progression of his chronic kidney disease, but he is not receiving it	96 (19.4)
The patient needs a drug treatment, a phosphate binder (calcium, sevelamer or lanthanum carbonate), because his serum phosphate is higher than normal values for a patient with chronic kidney disease despite an appropriate diet	60 (12.1)
The patient requires drug therapy with vitamin D (cholecalciferol, calciferol) because his serum 25(OH)D < 75 nmol/L, and he is in stage 3 or 4 CKD	82 (16.6)
The patient requires drug therapy with vitamin D (calcitriol or alfacalcidol) because he has hyperparathyroidism	100 (20.2)
The patient needs hypoglycaemic drug therapy because his glycated hemoglobin (HbA1c) is > 7% despite an appropriate diet	12 (2.4)
The patient needs drug therapy with sodium polystyrene sulfonate to treat is hyperkalemia (K + > 5.5 mmol/L)	3 (0.6)

*CKD* chronic kidney disease, *DRP* Drug Related Problem

144 DRPs were found, with an average of 1.4 ± 1.3 DRP per patient according to PCNE. 73.1% of patients had at least 1 DRP. While the majority of DRPs were related to drug selection, only 4.9% were related to dose selection (Table 3).

**Table 3:** Prevalence of DRP according to PCNE for 104 CKD patients (N = 144)

Drug-related problem	N (%)
Drug selection	137 (95.1)
Inappropriate drug according to guidelines/formulary	8 (5.6)
No indication for drug	55 (38.2)
Inappropriate combination of drugs, or drugs and herbal medications, or drugs and dietary supplements	26 (18.1)
No or incomplete drug treatment in spite of existing indication	48 (33.3)
Dose selection	7 (4.9)
Drug dose of a single active ingredient too high	7 (4.9)

*CKD* chronic kidney disease, *DRP* Drug Related Problem, *PCNE* Pharmaceutical Care Network Europe

### Translation stage

A panel of experts evaluated the items in the criterion based on their intelligibility, service of purpose, differentiation, and cultural suitability using the translation evaluation form. Each item was evaluated by a measurement level of 1–4 points. CVI score was found to be 0.95.

### Reliability analysis stage

A clinical pharmacist re-evaluated the pharmacotherapy of 30 randomly selected patients 1 month later, applied the test-retest method, and calculated correlation coefficient. The correlation coefficient value (p) was found to be 0.8.

### Conceptual validity stage

The pharmacotherapy plan of 104 patients was evaluated by the clinical pharmacist using both the PAIR criteria and the PCNE. There was a statistically significant difference between the prevalence of DRPs according to PAIR criteria (100.0%), and PCNE (73.1%) (p < 0.001).

## Discussion

The present study aimed to validate the use of PAIR in Turkish. On average, there were 4.8 ± 1.2 DRPs per patient according to PAIR criteria. In 2 studies conducted using the PAIR criteria the prevalence of the original developer DRP by Desrochers et al was found to be 21%, while the prevalence of the Brazilian version DRP by Marquito et al was 80% [17, 18]. The most significant reason for the high prevalence of DRP in our study may be that only dialysis patients were included in the study. Although studies on DRP in outpatient dialysis patients are limited, a recently published meta-analysis study analyzing DRPs in hospitalized patients found that DRP prevalence in CKD patients ranged from 12% to 87% [24]. A study by Liu et al. [25] found DRP in 77% of patients with chronic kidney disease receiving dialysis, and a study by Holm et al. [26] found it in 62%.

Regarding PAIR validation, the reliability found was considered good, with results indicating a moderate to perfect agreement between the DRPs found in the test-retest by the same evaluator. The correlation coefficient value found in our study is like the results of other studies [17, 18].

Regarding conceptual validity, our results revealed that the PAIR was able to identify clinically significant DRPs in patients with CKD. In our study, conceptual validity was evaluated with a different method than other studies. In the study by Marquito et al. [18], the DRP rates found by a nephrologist’s clinical judgment and a pharmacist using the PAIR criterion were compared. In our study, a clinical pharmacist compared DRPs using both the PAIR and the PCNE criteria. According to the nephrologist’s clinical judgment, the number of DRPs detected was higher than the number of DRPs detected by the pharmacist with PAIR in the other studies [17, 18]. In our study, the number of DRPs identified by PAIR was significantly higher than the number of DRPs determined by a clinical pharmacist’s clinical judgment (PCNE).

Although DRP was detected by all of the patients according to the PAIR criteria in this study, it was detected by 74% of the patients according to the PCNE classification. When pharmacotherapy plans are evaluated based on explicit criteria (like PAIR), more DRPs are likely to be identified than if they are evaluated based on implicit criteria (like PCNE). Only 67 DRPs were in both PAIR and PCNE evaluation.

The most prevalent categories were “needs drug treatment (angiotensin-converting enzyme inhibitor or angiotensin receptor blocker)” (19.4%), “requires drug therapy with vitamin D” (16.6%) and “needs a drug treatment, a statin” (14.72%) according to PAIR criteria. In the Canadian study, most of the DRPs were related to “non-optimal treatment adherence”, whereas in the Brazilian version, they were related to “interaction and drug have taken inadequately” [17, 18].

In this study, the lack of statin therapy was one of the three top causes of DRPs based on the PAIR criteria. However, there were no patients with a “statin indication” in the PCNE classification, which was used for conceptual validity and was based on the clinical evaluation of the clinical pharmacist. Because the KDIGO guideline does not recommend initiating statins or statin/ezetimibe combinations in adults with dialysis-dependent CKD [27].

Of the 144 DRPs detected by PCNE, 77 (53.5%) were not included in the PAIR criteria. These were mostly (36/77, 46.7%) caused using proton pump inhibitors (PPIs) without indication. This was followed by contraindicated drug use (such as silodosin, and trimetazidine) and high-dose drug use (such as escitalopram, and pitavastatin) (12/77, 15.6%). These results show that the PAIR criterion should be updated regularly.

The present study had several limitations. Firstly, the study included only patients’ prescription records. Data on the use of dietary supplements without a prescription could not be collected. Additionally, the study was conducted on dialysis patients, so the results cannot be generalized to all older adults with CKD.

There are also strengths to the method used in this study. Due to the number of patients and limited examination time showing a negative relationship with the number of physicians in Turkey, the use of explicit criteria may reduce the frequency of DRPs.

## Conclusion

Although this study shows that PAIR is easy to use and reliable and its use has been validated in Turkey, it is difficult to interpret it as an adequate tool for the evaluation of clinically significant DRPs in hemodialysis patients. By incorporating this instrument into pharmaceutical care in nephrology services and updating it at regular intervals, it may be possible to standardize and systematize data.

## Data Availability

The data that support the findings of this study are not openly available due to reasons of sensitivity and are available from the corresponding author upon reasonable request. Data are located in controlled access data storage at the Ethics Committee of the University Ankara. Example from: http://arastirma.medicine.ankara.edu.tr/etik-kurullar/
